# Motive Satisfaction Among Patients with Chronic Primary Pain: A Replication

**DOI:** 10.1007/s10880-023-09942-8

**Published:** 2023-02-18

**Authors:** Alina Scheidegger, Juan Martín Goméz Penedo, Larissa Tatjana Blättler, Selma Aybek, Nina Bischoff, Martin grosse Holtforth

**Affiliations:** 1 Psychosomatic Medicine, Department of Neurology, Inselspital, Bern University Hospital, University of Bern, Bern, Switzerland; 2 Facultad de Psicología, CONICET Universidad de Buenos Aires, Buenos Aires, Argentina; 3 Department of Psychology, University of Bern, Bern, Switzerland

**Keywords:** Motive satisfaction, Motivational incongruence, Psychological distress, Chronic pain, Interdisciplinary multimodal pain treatment

## Abstract

We set out to replicate findings of significant (a) reductions in pain, psychological distress, and motivational incongruence (i.e., insufficient motive satisfaction) after interdisciplinary multimodal pain treatment and (b) associations between reductions in motivational incongruence (i.e., improved motive satisfaction) and decreases in psychological distress (Vincent et al., Journal of Clinical Psychology in Medical Settings 28:331–343, 2021). 475 Patients with chronic primary pain completed standardized self-reported questionnaires assessing motivational incongruence, psychological distress, pain intensity, and pain interference at intake and discharge from a tertiary psychosomatic university clinic. We used hierarchical linear models to analyze motivational incongruence’s effects on psychological distress. We partially replicated Vincent et al.’s findings. Significant reductions in pain, psychological distress, and motivational incongruence after treatment were found. Reductions in motivational incongruence were associated with reductions in psychological distress. Similarly, a better motive satisfaction mediated the relationship between pain interference and psychological distress. Our findings show that reducing motivational incongruence may be a key component of treating chronic primary pain; we recommend to assess and target motivational incongruence to improve interdisciplinary multimodal pain treatment.

## Introduction

In his Consistency Theory, which is grounded in basic psychological research and research on general processes and mechanisms of change in psychotherapy, Klaus Grawe (2004) took a motivational perspective to explain the development and maintenance of mental disorders. According to this theory, individuals strive for the “compatibility of many simultaneously transpiring mental processes” (i.e., *consistency*; Grawe, 2007, p. 170), and he considered need satisfaction (i.e., *congruence*) as the central component of consistency. Thus, congruence is achieved when an individual’s psychological needs (attachment, control, self-esteem enhancement, pleasure) and perceived reality align (Caspar & grosse Holtforth, 2010; Grawe, 2004, 2007).

Accordingly, (motivational) incongruence describes the degree of discrepancy between a person’s motives and perceived reality. Assumedly, individuals strive to reduce the level of incongruence if their motives and related behaviours do not achieve satisfying their needs. In other words, the striving for congruence serves individuals to satisfy their needs via individual motives and behaviours, fostering adaptive functioning and psychological health (Fries & Grawe, 2006).

Motives can be further differentiated into approach and avoidance motives. Approach motives target desired experiences as instances of need satisfaction, whereas avoidance motives target undesired experiences resulting from need violations (Westermann et al., 2019). Therefore, approach incongruence is experienced when motives and perceived reality diverge, and avoidance incongruence is experienced when unpleasant experiences that conflict with personal motives cannot be avoided. The subjective perception of motivational incongruence can be measured with the validated Incongruence Questionnaire (INC) or its short form INC-S, measuring both approach and avoidance incongruence that is particularly suitable for clinical use (grosse Holtforth et al., 2004).

In Europe, 19% of adults suffer from chronic pain that has lasted for at least 3 months or is recurrent (Breivik et al., 2006). Chronic pain causes considerable suffering, affects almost every aspect of a person’s life, and is often accompanied by depression and anxiety (Velly & Mohit, 2018). 20–50% Of patients with chronic pain suffer from co-morbid depression and 25% of chronic pain patients suffer from anxiety (Knaster et al., 2012; Mills et al., 2019). Chronic pain and psychological disorders seem closely related, often co-occur, and may mutually precede and/or increase each other over time (Velly & Mohit, 2018). Accordingly, higher depression and anxiety levels are often accompanied by more severe pain in several locations (Angst et al., 2020; Gómez Penedo et al., 2020; Velly & Mohit, 2018). Furthermore, longitudinal data shows that reducing depressive and anxiety symptoms may go along with reducing pain and vice versa (Angst et al., 2020; Gerrits et al., 2015).

The biopsychosocial model of pain emphasizes the multifactorial conceptualization of coping with chronic pain, quality of life, the development of disability (Turk & Okifuji, 2002), and potential chronification (Kendall, 1999). Apart from somatic factors, cognitive (e.g., fear-avoidance beliefs and attitudes), behavioural (e.g., pain catastrophizing, somatization, coping styles), as well as various social variables (e.g., positive and negative social reinforcement by the environment, daily activities, family life, cultural factors) are often considered to understand what influences the development and chronification of chronic pain (Dorner et al., 2018; Hruschak & Cochran, 2018; Nieminen et al., 2021).

With its motivational focus, Consistency Theory is taken as an integrative theoretical foundation that allows for incorporating disorder-specific, as well as transdiagnostic theories and related interventions into psychotherapy (Grawe, 2004). Motivational incongruence is assumed to lead to developing and maintaining psychological distress and mental disorders (Fries & Grawe, 2006). Thus, conceptualizing chronic pain in terms of incongruence may aid in improving our knowledge of chronic pain and its treatment by placing it within a broader conceptual and therapeutic context.

Some research on chronic pain has used a similar concept as incongruence by examining personal goal frustration. More specifically, Vervoort & Trost (2017) showed that chronic pain can interfere with personal goals regarding physical integrity or identity-related goals. Furthermore, many patients with chronic pain experience a partial or total loss of previous social roles in various domains, resulting from disengaging and withdrawing from previously valued activities to avoid pain, also leading to higher depression scores (Harris et al., 2003; Vlaeyen & Linton, 2012). In incongruence terms, these findings can be interpreted in that patients with chronic pain experience higher levels of incongruence regarding both pain-related and more general goals. Empirically, high levels of psychological symptoms such as anxiety and depression and low levels of well-being have been linked to increased levels of motivational incongruence (Brockmeyer et al., 2015; Kelly et al., 2015). Furthermore, psychological distress and mental disorders can be sources of incongruence themselves (Westermann et al., 2019). A reduced incongruence after treatment has generally been linked to better therapeutic outcomes, especially when approach goals are met rather than avoidance goals (Berking et al., 2003; grosse Holtforth et al., 2005; grosse Holtforth, 2008; Wollburg & Braukhaus, 2010). Since goal adjustment seems to increase the quality of life and well-being among patients with chronic pain (Esteve et al., 2018; Ramírez-Maestre et al., 2019), redefining and engaging in new realistic goals might reduce perceived motivational incongruence.

To the best of our knowledge, Vincent et al. (2021) have been the first to directly investigate the relationship between chronic pain and motivational incongruence as well as respective associations with psychological distress using the INC-S. They hypothesized that patients with chronic pain have higher levels of motivational incongruence than a healthy norm sample and that pain interference and motivational incongruence can be reduced after interdisciplinary multimodal pain treatment (Vincent et al., 2021). Accordingly, Vincent et al. (2021) could show that patients with chronic pain in a interdisciplinary multimodal pain treatment have higher levels of motivational incongruence at intake than a healthy norm sample. In addition, motivational incongruence and psychological distress were significantly reduced over treatment. Furthermore, the satisfaction of approach motives seemed to mediate the relationship between pain interference, and psychological distress. These results suggest that reductions in motivational incongruence during inpatient treatment may have contributed to reducing the level of psychological distress perceived by patients.

The present study aims to replicate the findings of Vincent et al. (2021) using a separate and larger sample of patients with chronic primary pain receiving an inpatient interdisciplinary multimodal pain treatment at the same site. It was hypothesized that patients with chronic primary pain experience higher levels of motivational incongruence at intake than a healthy norm sample. In addition, it was expected that the treatment would significantly reduce both motivational incongruence and psychological distress across treatment. In contrast to Vincent et al. (2021), who used multiple regression analyses, this study will use hierarchical linear models (HLM) to replicate the association of psychological distress with motivational approach and avoidance incongruence. Using HLM will allow for differentiating within and between patient effects. As in Vincent et al. (2021), exploratory analyses will be conducted to replicate the changes in the different incongruence dimensions throughout treatment. Lastly, it is expected that the better satisfaction of approach motives and, therefore, better approach incongruence will mediate the effect of change in pain interference on change in psychological distress.

## Methods

### Sample

475 Patients with chronic primary pain received inpatient care in a tertiary psychosomatic university clinic between December 2015 and February 2022. All patients fulfilled the diagnostic criteria of chronic primary pain (MG30.0) according to the ICD-11 (World Health Organization, 2019). Therefore, patients with primary chronic pain suffer from chronic pain in one or more anatomical regions that persists for more than three months or is recurrent and experience significant emotional distress and/or functional disability (World Health Organization, 2019). Moreover, the symptoms should not be better accounted for by any other diagnosis, indicating secondary pain syndromes, e.g., chronic cancer pain (Nicholas et al., 2019). Individuals younger than 18 years, with insufficient German-language proficiency, and/or refusing general consent to further use their data were excluded.

### Norm Sample

Means and standard deviations of a norm sample described by grosse Holtforth et al. (2004) were used to compare the degree of incongruence among inpatients with a healthy norm. This norm sample consisted of data from 707 healthy individuals from various projects of the authors in the social environment of (under-)graduate students. Individuals in this norm sample were on average 40.2 years old (*SD* = 15.1 years; range = 18–87 years old), and 60.9% were female.

### Procedures

All patients completed psychometric assessments at intake and discharge of the interdisciplinary multimodal pain treatment program for quality management purposes. During three 45-min psychometry sessions, patients completed a battery of self-reported questionnaires with the assistance of a research assistant. The INC-S was part of this battery, which included questionnaires on the patient’s overall condition, psychopathological symptoms, clinically relevant behaviour and experience, as well as other treatment-related questions.

### Interdisciplinary Multimodal Pain Treatment

Interdisciplinary multimodal pain treatment requires that psychiatric, psychosomatic or psychological disciplines treat patients with chronic pain for at least 7 days. Further, at least three active therapy methods must be used in patient-specific combinations: psychotherapy, physiotherapy, relaxation procedures, occupational therapy, medical training therapy, workplace training/training for everyday living, and interventional pain therapy. The duration of therapy per week is at least 540 min. Treatment was adapted to the needs of the patients, but most patients received a range of treatments: psychotherapy, medical interventions, pharmacotherapy, physiotherapy, and occupational therapy (Arnold et al., 2014). On average, patients stayed for 24 days (*M* = 24.08 days; *SD* = 4.72 days).

### Measures

In accordance with the Initiative on Methods, Measurement, and Pain Assessment in Clinical Trials (IMMPACT) recommendations (Dworkin et al., 2005; Turk et al., 2008), as well as the VAPAIN consensus statement (Kaiser et al., 2018), we defined a decrease in pain intensity, pain interference, and psychological distress as primary outcome measures for this research.

*BPI:* To measure pain intensity and pain interference, the German version of the Brief Pain Inventory (BPI) was used (Radbruch et al., 1999). Pain intensity is assessed with four items regarding the worst, least, average, and current pain with a Likert scale from 0 to 10, ranging from no pain at all (0) to the worst pain imaginable (10). Pain interference was assessed with seven Likert scale items, ranging from no interference (0) to complete interference (10). Both subscales were calculated by averaging the items, leading to a possible score of 0–10 for both subscales. The translated version of the BPI has shown good psychometric properties and confirmed the two-factor structure of the BPI (Radbruch et al., 1999). In this sample, Cronbach’s alpha for pain intensity was 0.88, and for pain-related inference was 0.85, which can be considered good. According to the IMMPACT criteria, a more than 30% reduction in pain intensity can be regarded as an at least moderate clinically relevant decrease during treatment, found in 16% of this sample patients. However, 58.3% of all patients reported a clinically significant reduction in pain interference across treatment, measured by a one-unit decrease on the NRS scale (Dworkin et al., 2005).

*HADS-D*: The German version of the Hospital Anxiety and Depression Scale (HADS-D) was used to assess psychological distress during the past week (Petermann, 2011). The questionnaire consists of seven items each for the subscales anxiety and depression. All items are rated on four-point Likert scales from 0 to 3, leading to a possible score of 0–21 for each subscale. A total score for psychological distress can be calculated by adding the subscales. The German version of the HADS-D has good psychometric properties, and the two-factor structure has been confirmed repeatedly (Herrmann-Lingen et al., 2011; Petermann, 2011). The internal consistency for this sample was good, with Cronbach’s alpha of 0.87. According to the reliable change index (RCI), 130 (27.3%) of all patients reported clinically significant psychological distress changes from pre- to post-treatment.

*INC-S*: The discrepancy between the patient’s motives and perceived reality (incongruence) was measured using the validated German short version of the Incongruence Questionnaire (INC-S) (grosse Holtforth et al., 2004). The original INC has fourteen subscales for approach incongruence and nine for avoidance incongruence, which can be summarized in summary scores of approach incongruence, avoidance incongruence, and a total incongruence score. The short questionnaire consists of those 23 items that had shown the highest item-total correlation with each of the respective subscales of the original version in different patient samples. Therefore, each of the INC-S items can be considered a proxy for the original subscale (grosse Holtforth et al., 2004). Accordingly, fourteen items can be summarized for computing the scale for the satisfaction of approach motives (e.g.,” I’ve been in control of myself”) ranging from low (1 = not at all) to high motive satisfaction (5 = a lot). Nine items represent the subscale for the satisfaction of avoidance motives (e.g., “I’ve been criticized”), measuring the degree of experiencing aversive transactions ranging from high incongruence (5 = very often) to low incongruence (1 = hardly ever). The scale score for approach incongruence is computed by averaging the inverted approach-item ratings, and the avoidance incongruence score is determined by averaging the avoidance-item ratings. The total incongruence score is calculated by averaging the approach and avoidance incongruence scores (grosse Holtforth et al., 2004). Reliability analyses in previous studies showed good internal consistency scores for approach incongruence of 0.91 and avoidance incongruence of 0.87 in an inpatient sample. In the current sample, Cronbach’s alpha for approach incongruence was 0.86 and avoidance incongruence 0.76, which can be considered good.

### Statistical Analyses

R and IBM SPSS Statistics (version 27) were used for statistical analyses (IBM Corp, 2020; R Core Team, 2021). Descriptive analyses were first computed for the demographic and clinical data. The means of the patients with chronic primary pain and a norm sample (grosse Holtforth et al., 2004) were compared using *t*-tests to determine if the incongruence scores differ for total incongruence, approach incongruence, avoidance incongruence, as well as all single items/scales of the INC-S. Paired *t*-tests were conducted to assess the changes between intake and discharge. The association of the variables under investigation was measured by computing Pearson correlations. The significance level was set at α = 0.05 (two-tailed).

To test the effects of incongruence (approach and avoidance motives) on psychological distress (HADS-D total score), hierarchical linear models (HLM; Goméz Penedo et al., 2019; Raudenbush & Bryk, 2002) were computed to handle the hierarchical structure of the data (repeated assessment of outcome variables being “nested” within the patients). Other than Vincent et al. (2021), we used HLMs, because this allows the differentiation of effects within patients (the effects of variations in approach and avoidance motives on psychological distress) and between patients (the effects of the overall level of approach and avoidance motives on psychological distress).

Since three measurement points are recommended for HLM, the statistical procedures needed to be slightly modified to adjust for the two measurement points at intake and discharge (Barnett et al., 1993; Pfeifer et al., 2018). Therefore, an approach usually applied in couple’s research was used, having two measurements from one person of the couple (Smith & Sayer, 2013). For this, the items of the outcome variables were matched based on their variance and randomly assigned to two scales, forming two parallel and equivalent subscales for each outcome variable. This resulted in having two measures at each time point for each outcome variable, providing enough variability to run two-level hierarchical linear models.

A fully unconditional model was calculated for psychological distress in the first step. Then, a time-as-only predictor model (TAOP) with time centered at intake was computed as an additional measure of change in psychological distress. Next, a conditional hybrid random effect model was calculated for psychological distress, including level-1-predictors of the variation of the patients around their own mean of approach and avoidance motives (person-mean centered) and level-2-predictors of the mean value over both measurement points (grand-mean centered) (Falkenström et al., 2017). In a fourth model, a conditional hybrid random effect model was needed to address time effects due to the significant TAOP model, adjusting for time as an additional level-1 predictor. Lastly, age, sex, pain duration, intensity, and interference were added as level-2 predictors (grand-mean centered). The same HLM models were calculated to test the effects of psychological distress (HADS-D total) on the approach and avoidance motive satisfaction to test the assumed causal relationship between psychological distress and motive satisfaction.

The predictive value of the single-item approach and avoidance incongruence on psychological distress at post-treatment was investigated exploratorily using a linear regression analysis with a stepwise elimination technique using the same control and pain-related variables as in the HLM.

A final mediation model was computed to investigate the change of motivational incongruence in approach and avoidance motives throughout the treatment as a mediator of the effect of change in pain interference on change in psychological distress using bootstrapping (Preacher & Hayes, 2008). Intercepts and change estimates were computed for pain interference, psychological distress, approach, and avoidance incongruence. Age, sex, and pain duration were included as control covariates.

## Results

### Descriptive Statistics: Demographic and Clinical Data

Table 1 summarizes all socio-demographic and pain-related data. On average, patients were 47.92 years old, and the sample consisted mostly of women. Most of the patients were married or in a romantic relationship and had completed an apprenticeship as their highest level of education. More than 40% of the patients reported suffering from pain between 1 and 5 years; about one-third for more than 10 years. More than two-thirds of the patients reported partial or total pain-related, medically certified inability to work. In Switzerland, the certified inability to work can be partial or complete. The related percentage indicates to which degree people can no longer carry out their previous activity due to the pain condition or the risk of worsening their state of health.

**Table 1:** Demographic and clinical characteristics

			Range
Age—*M* (*SD*)	47.92	(13.95)	18**–**81
Sex—*N* (%)			
Female	302	(63.6)	
Male	173	(36.4)	
*Marital status—N (%)*			
In a relationship	53	(11.2)	
Married	207	(43.9)	
Divorced/separated	106	(22.5)	
Widowed	15	(3.2)	
Single	91	(19.3)	
*Education—N (%)*			
Compulsory school not completed	8	(1.7)	
Compulsory school	86	(18.3)	
Apprenticeship	255	(54.1)	
High School	24	(5.1)	
Community college degree	63	(13.4)	
University or college degree	35	(7.4)	
*Pain duration—N (%)*			
0**–**3 months	4	(0.9)	
4**–**6 months	20	(4.3)	
7**–**11 months	25	(5.3)	
1**–**5 years	209	(44.5)	
6**–**10 years	70	(14.9)	
> 10 years	142	(30.2)	
*Inability to work—N (%)*			
0%	166	(35.5)	
≤ 25%	7	(1.5)	
≤ 50%	29	(6.2)	
≤ 75%	18	(3.9)	
≤ 100%	247	(52.9)	

Age (*N* = 475); sex (*N* = 475); marital status (*N* = 472); education (*N* = 471); pain duration (*n* = 470); inability to work (*N* = 467)

*N* Number of patients*; M* mean; *SD* standard deviation

### Pre-treatment Analysis

Pearson correlations were calculated to determine the relationship between the predictor and outcome variables and are shown in Table 2. Findings suggest that higher levels of motivational incongruence were significantly associated with higher pain intensity, pain interference, and psychological distress.

**Table 2:** Correlations of study variables

	1	2	3	4	5	6	7	8	9	10	11
Total incongruence INC-S pre-treatment	–										
Approach incongruence INC-S pre-treatment	.950***	–									
Avoidance incongruence INC-S pre-treatment	.859***	.656***	–								
Pain intensity BPI pre-treatment	.139***	.099***	.173***	–							
Pain interference BPI pre-treatment	.482***	.461***	.409***	.481***	–						
Psychological distress HADS-D pre-treatment	.654***	.634***	.543***	.217***	.549***	–					
Total incongruence INC-S post-treatment	.725***	.688***	.624***	.132***	.386***	.541***	–				
Approach incongruence INC-S post-treatment	.699***	.702***	.536***	.126***	.384***	.532***	.951***	–			
Avoidance incongruence INC-S post-treatment	.610***	.514***	.631***	.114***	.304***	.437***	.861***	.661***	–		
Pain intensity BPI post-treatment	.146***	.108***	.175***	.695***	.389***	.188***	.197***	.191***	.164***	–	
Pain interference BPI post-treatment	.453***	.412***	.418***	.407***	.566***	.427***	.590***	.552***	.522***	.538***	–
Psychological distress HADS-D post-treatment	.597***	.554***	.535***	.226***	.460***	.713***	.729***	.685***	.641***	.321***	.658***

*INC-S* incongruence questionnaire—short version, *BPI* brief pain inventory—German version, *HADS-D* hospital anxiety and depression scale—German version

*p < .05, **p < .01, ***p < .001

Means, standard deviations, and *t*-tests comparing all incongruence items, avoidance, approach and total incongruence of the patients with chronic primary pain with a norm sample are shown in Table 3. The studied patients with chronic primary pain had significantly higher incongruence scores than the norm sample in approach, avoidance, and total incongruence. All single INC-S items except for *Status*, *Appreciation/Approval*, and *Intimacy/Attachment* had significantly different incongruence scores with effect sizes ranging mostly between medium to high. Whereas almost all significant differences showed higher mean incongruence scores among patients with chronic primary pain, the values of the approach items for *Receiving Help* and *Altruism*, as well as the avoidance-item of *Hurting Others* were significantly lower among patients with chronic primary pain than among subjects in the norm sample.

**Table 3:** One-sample *t* test comparing means between patients with chronic primary pain at pre-treatment and a norm sample for incongruence subscales of the INC-S

	Patients with chronic primary pain Pre-treatment		Healthy control group		*t*	*d*
	*M*	*SD*	*M*	*SD*		
Achievement/performance	3.49	1.34	2.21	0.78	20.71***	1.168
Autonomy	2.90	1.39	2.05	0.77	13.47***	0.756
Trust in oneself	2.82	1.33	2.10	0.78	11.71***	0.660
Status	2.74	1.42	2.64	0.76	1.56	0.088
Appreciation/approval	2.04	1.18	2.03	0.66	0.19	0.010
Affiliation/sociability	2.89	1.47	2.46	1.01	5.96***	0.341
Self-reward	2.76	1.35	2.50	0.90	3.97***	0.227
Control	2.42	1.25	2.22	0.74	3.45***	0.195
Intimacy/attachment	2.55	1.59	2.46	1.18	1.12	0.064
Receiving Help	2.08	1.25	2.50	0.85	6.88***	0.393
Education/understanding	2.94	1.38	2.37	0.70	9.34***	0.521
Belief/sense of meaning	2.61	1.33	2.26	0.80	5.64***	0.319
Altruism	2.12	1.30	2.37	0.84	4.02***	0.228
Excitement/diversion	3.37	1.33	2.33	0.85	16.40***	0.931
Weakness/loss of control	3.20	1.35	2.59	0.82	9.65***	0.546
Failure	3.06	1.36	1.82	0.74	20.20***	1.133
Blame/criticism	2.23	1.25	2.04	0.86	3.25**	0.177
Dependence/loss of autonomy	2.45	1.34	1.88	0.76	9.30***	0.523
Helplessness	3.19	1.29	2.04	0.87	18.31***	1.045
Humiliation/embarrassment	1.99	1.24	1.53	0.60	8.50***	0.472
Hurting others	1.86	1.14	1.97	0.76	1.99*	0.114
Separation/being alone	2.19	1.35	1.99	0.86	3.11**	0.177
Not being respected/accepted	2.08	1.25	1.67	0.72	7.14***	0.402
Total incongruence INC-S	2.61	0.72	2.13	0.51	13.41***	0.769
Approach incongruence INC-S	2.70	0.80	2.32	0.54	9.75***	0.557
Avoidance incongruence INC-S	2.47	0.76	1.95	0.59	13.20***	0.764

N1 475, N2 707, *M* mean, *SD* standard deviation, *t t* value, *d* Cohen’s *d,* *INC-S* Incongruence questionnaire—short version

*p < .05, **p < .01, ***p < .001

### Comparisons Between Pre‑ and Post‑Treatment

Mean values and standard deviations for different outcome measures at intake and discharge are shown in Table 4. Paired *t*-tests showed that all outcome measures improved significantly over 3 weeks. Table 5 shows all INC-S items’ mean values, standard deviation, *t*-tests, and effect sizes (as proxies of the INC scales). After treatment, patients reported lower incongruence regarding all single items with considerable variations for both approach and avoidance motives. Whereas the largest pre-post differences were found for the approach items of *Achievement/Performance,* and the avoidance items of *Helplessness*; *Intimacy/Attachment,* and *Weakness/Loss of Control* showed the smallest pre-post differences for approach items and avoidance items, respectively.

**Table 4:** Number of patients, mean, standard deviation, pre-post comparison, and effect size of different outcome measures

	*N*	Pre-treatment		Post-treatment		*t*	*d*
		*M*	*SD*	*M*	*SD*		
Total incongruence INC-S	475	2.06	0.717	2.28	0.725	26.66***	0.612
Approach incongruence INC-S	475	2.70	0.800	2.37	0.806	22.68***	0.520
Avoidance incongruence INC-S	475	2.47	0.756	2.14	0.765	22.23***	0.510
Psychological distress HADS-D	475	19.93	8.188	14.88	8.314	35.17***	0.807
Pain intensity BPI	475	5.35	1.690	5.07	1.886	8.62***	0.198
Pain interference BPI	475	5.73	1.921	4.43	2.019	30.83***	0.707

*N* number of patients; *M* mean; *SD* standard deviation; *t t* value; *d* Cohen’s *d*; INC-S: Incongruence Questionnaire – Short Version, *BPI* Brief Pain Inventory—German version; *HADS-D* Hospital Anxiety and Depression Scale—German version

*p < .05, **p < .01, ***p < .001

**Table 5:** Dependent *t*-tests of the incongruence questionnaire (INC-S) items between pre- and post-treatment

		*N*	Pre-treatment		Post-treatment		*t*	*d*
			*M*	*SD*	*M*	*SD*		
Achievement/performance	1	475	3.49	1.34	2.89	1.24	19.28***	0.442
Autonomy	2	475	2.90	1.39	2.54	1.25	11.82***	0.271
Trust in Oneself	3	475	2.82	1.33	2.38	1.23	15.89***	0.365
Status	4	475	2.74	1.42	2.47	1.26	7.99***	0.183
Appreciation/Approval	5	475	2.04	1.18	1.84	1.01	8.18***	0.188
Affiliation/Sociability	6	475	2.89	1.47	2.36	1.33	16.71***	0.383
Self-Reward	7	475	2.76	1.35	2.42	1.25	11.27***	0.259
Control	8	475	2.42	1.25	2.22	1.17	7.14***	0.164
Intimacy/Attachment	9	475	2.55	1.59	2.48	1.61	2.05*	0.047
Receiving Help	10	475	2.08	1.25	1.87	1.08	7.48***	0.172
Education/Understanding	11	475	2.94	1.38	2.52	1.29	13.98***	0.321
Belief/Sense of Meaning	12	475	2.61	1.33	2.33	1.28	9.35***	0.214
Altruism	13	475	2.12	1.30	2.01	1.15	4.03***	0.092
Excitement/Diversion	14	475	3.37	1.33	2.90	1.30	15.36***	0.352
Weakness/Loss of Control	15	475	3.20	1.35	3.09	1.37	3.10**	0.071
Failure	16	475	3.06	1.36	2.48	1.29	17.67***	0.405
Blame/Criticism	17	475	2.23	1.25	1.87	1.07	12.37***	0.284
Dependence/Loss of Autonomy	18	475	2.45	1.34	2.27	1.30	5.45***	0.125
Helplessness	19	475	3.19	1.29	2.62	1.33	17.91***	0.411
Humiliation/Embarrassment	20	475	1.99	1.24	1.69	1.01	10.90***	0.250
Hurting Others	21	475	1.86	1.14	1.52	0.94	14.88***	0.341
Separation/Being Alone	22	475	2.19	1.35	1.92	1.22	8.76***	0.201
Not Being Respected/Accepted	23	475	2.08	1.25	1.78	1.05	10.64***	0.244

*N* number of patients, *M* mean; *SD* standard deviation, *t t* value, *d* Cohen’s *d*, INC-S Incongruence questionnaire—short version

*p < .05, **p < .01, ***p < .001

### Hierarchical Linear Modeling Predicting Psychological Distress

The results of all models are summarized in Table 6. The unconditional model showed an estimated average psychological distress score of 1.26 across treatment. The time-as-only-predictor model (TAOP) showed a significant negative time effect on psychological distress, indicating from intake to discharge, patients psychological distress decreased with 0.33 units. The model comparison of the TAOP and unconditional model indicated a significantly better fit for the TAOP model.

**Table 6:** Summary of the unconditional, time-as-only predictor, conditional random effect, and conditional random effect detrending models analyzing the effect of approach and avoidance incongruence on psychological distress

	Psychological distress		
	$\gamma$	*SE*	*t*
*Unconditional model*			
Intercept	1.26	0.03	50.19***
*Time-as-only predictor model*			
Intercept	1.09	0.03	40.73***
Time	− 0.33	0.02	− 17.54***
Model comparison	$\Delta {\chi }^{2}\left(3\right)$ = 543.55, *p* < .001		
*Conditional random effect model*			
Intercept	− 0.19	0.07	− 2.89**
Between patient effect for approach incongruence	0.37	0.03	11.17***
Within patient effect for approach incongruence	0.35	0.04	9.10***
Between patient effect for avoidance incongruence	0.22	0.04	6.22***
Within patient effect for avoidance incongruence	0.19	0.04	5.12***
Model comparison	$\Delta {\chi }^{2}\left(6\right)$ = 314.96, *p* < .001		
*Conditional random effect model detrending*			
Intercept	− 0.30	0.07	− 4.62***
Time	− 0.22	0.02	− 12.54***
Between patient effect for approach incongruence	0.37	0.03	11.14***
Within patient effect for approach incongruence	0.23	0.03	6.61***
Between patient effect for avoidance incongruence	0.22	0.04	6.30***
Within patient effect for avoidance incongruence	0.09	0.03	2.84**
Model comparison	$\Delta {\chi }^{2}\left(1\right)$ = 144.39, *p* < .001		
*Conditional random effect model detrending*			
Intercept	− 0.44	0.07	− 6.59***
Time	− 0.17	0.02	− 9.36***
Between patient effect for approach incongruence	0.34	0.03	10.70***
Within patient effect for approach incongruence	0.20	0.03	6.20***
Between patient effect for avoidance incongruence	0.20	0.03	5.86***
Within patient effect for avoidance incongruence	0.08	0.03	2.52*
Pain intensity	0.03	0.01	4.80***
Pain interference	0.03	< 0.01	5.35***
Model comparison	$\Delta {\chi }^{2}\left(2\right)$ = 63.19, *p* < .001		
*Conditional random effect model detrending*			
Intercept	− 0.49	0.11	− 4.46***
Time	− 0.17	0.02	− 9.37***
Between patient effect for approach incongruence	0.34	0.03	10.74***
Within patient effect for approach incongruence	0.2	0.03	6.21***
Between patient effect for avoidance incongruence	0.2	0.03	5.84***
Within patient effect for avoidance incongruence	0.08	0.03	2.52*
Pain intensity	0.03	0.01	4.75***
Pain interference	0.03	< 0.01	5.34***
Age	< 0.01	< 0.01	0.63
Sex	0.01	0.03	0.18
Pain duration	< 0.01	< 0.01	0.68
Model comparison	$\Delta {\chi }^{2}\left(3\right)$ = 0.87, *p* = .83		

$\gamma$ Regression coefficient; *SE* standard error; *t* *t* value

*p < .05, **p < .01, ***p < .001

For the conditional hybrid random effect model, person-mean centered approach and avoidance incongruence scores were included as level-1 predictors and average levels of approach and avoidance incongruence scores as level-2 predictors of the intercept representing the average psychological distress. Significant between patient effects of approach incongruence and avoidance incongruence could be found for psychological distress, as well as significant within patient effects of approach incongruence and avoidance incongruence. A one-unit decrease in the sample’s approach incongruence mean score was associated with a reduction of 0.37 in psychological distress. On the other hand, a one-unit decrease in the sample’s avoidance incongruence mean was associated with a reduction of 0.22 in psychological distress. A one-unit decrease from the patient’s own mean was associated with a reduction of 0.35 in psychological distress for approach incongruence and 0.19 in psychological distress for avoidance incongruence. The model comparison of the conditional hybrid random effect model and TAOP indicated a significantly better fit for the conditional hybrid random effect model.

Significant between and within patient effects of approach incongruence and avoidance incongruence could still be found when controlled for time in the detrended model to further replicate the conditional model. The model comparison of the conditional random effect model detrending and conditional hybrid random effect model indicated a significantly better fit for the detrending model.

In an additional model, pain intensity and interference were included as level-2 predictors to control for the effect of pain intensity and interference on psychological distress. Significant effects of time, between and within patient effects of approach incongruence and avoidance incongruence could still be found for psychological distress. This model, including pain intensity and pain interference as level-2 predictors fitted the data significantly better than the model without.

A second additional model controlling for age, sex, and pain duration as level-2 predictors did not fit the data better than the detrending model, including pain intensity and pain interference as additional level-2 predictors.

### Hierarchical Linear Modeling Predicting Approach and Avoidance Incongruence

Due to the assumed relationship between reduced psychological motive satisfaction and distress, additional HLMs were defined to test the association of approach incongruence and the change in psychological distress from pre- to post-treatment, respectively, avoidance incongruence and the change in psychological distress. Detailed information on the models can be found in Table 7.

**Table 7:** Summary of the unconditional, time-as-only predictor, conditional random effect, and conditional random effect detrending models analyzing the effect of psychological distress on approach and avoidance incongruence

	Approach Incongruence			Avoidance Incongruence		
	$\gamma$	*SE*	*t*	$\gamma$	*SE*	*t*
*Unconditional model*						
Intercept	2.53	0.03	74.56***	2.31	0.03	74.13***
*Time-as-only predictor model*						
Intercept	2.37	0.04	64.15***	2.11	0.03	61.22***
Time	− 0.32	0.03	− 11.34***	− 0.4	0.03	− 13.22***
Model comparison	$\Delta {\chi }^{2}\left(3\right)$ = 355.42, *p* < .001			$\Delta {\chi }^{2}\left(3\right)$ = 164.97, *p* < .001		
*Conditional random effect model*						
Intercept	1.33	0.06	22.27***	1.3	0.06	22.09***
Between patient effect for psychological distress	0.07	< 0.01	21.87***	0.06	< 0.01	18.64***
Within patient effect for psychological distress	0.05	< 0.01	12.07***	0.05	< 0.01	14.03***
Model comparison	$\Delta {\chi }^{2}\left(1\right)$ = 399.59, *p* < .001			$\Delta {\chi }^{2}\left(1\right)$ = 273.94, *p* < .001		
*Conditional random effect model detrending*						
Intercept	1.28	0.06	20.90***	1.19	0.06	19.21***
Time	− 0.11	0.03	− 3.96***	− 0.23	0.04	− 6.02***
Between patient effect for psychological distress	0.07	< 0.01	21.83***	0.06	< 0.01	18.61***
Within patient effect for psychological distress	0.04	< 0.01	8.25***	0.03	< 0.01	7.28***
Model comparison	$\Delta {\chi }^{2}\left(1\right)$ = 15.33, *p* < .001			$\Delta {\chi }^{2}\left(1\right)$ = 35.73, *p* < .001		
*Conditional random effect model detrending*						
Intercept	1.23	0.07	17.51***	1.15	0.08	15.40***
Time	− 0.10	0.03	− 3.39***	− 0.22	0.03	− 5.55***
Between patient effect for psychological distress	0.07	< 0.01	19.98***	0.05	< 0.01	15.90***
Within patient effect for psychological distress	0.04	< 0.01	7.69***	0.03	< 0.01	6.32***
Pain intensity	< 0.01	0.01	0.53	<—0.01	0.01	− 0.43
Pain interference	0.01	0.01	2.10*	0.03	0.01	2.65**
Model comparison	$\Delta {\chi }^{2}\left(2\right)$ = 5.34, *p* = .07			$\Delta {\chi }^{2}\left(2\right)$ = 7.02, *p* < .05		
*Conditional random effect model detrending*						
Intercept	1.66	0.14	12.27***	1.43	0.14	10.56***
Time	− 0.09	0.03	− 3.32***	− 0.22	0.04	− 5.50***
Between patient effect for psychological distress	0.07	< 0.01	19.81***	0.05	< 0.01	15.64***
Within patient effect for psychological distress	0.04	< 0.01	7.68***	0.03	< 0.01	6.26***
Pain intensity	0.01	0.01	0.74	<—0.01	0.01	-0.27
Pain interference	0.02	0.01	2.16*	0.03	0.01	2.69**
Age	− 0.01	< 0.01	− 3.24**	<—0.01	< 0.01	− 2.58*
Sex	− 0.09	0.05	− 1.81	− 0.04	0.05	− 0.88
Pain duration	< 0.01	< 0.01	− 1.13	< 0.01	< 0.01	0.80
Model comparison	$\Delta {\chi }^{2}\left(3\right)$ = 14.83, *p* < .01			$\Delta {\chi }^{2}\left(3\right)$ = 8.17, *p* < .05		

$\gamma$ regression coefficient; *SE* standard error; *t* *t* value

*p < .05, **p < .01, ***p < .001

For the prediction of approach incongruence, the model including age, sex, pain duration, pain intensity, and pain interference as additional level-2 predictors, fitted the data better than the model with only pain intensity and pain interference as additional level-2 predictors. Significant effects of time, between and within patient effects of psychological distress could be found for approach incongruence even when controlled for age, sex, pain intensity, pain interference and pain duration. The same could be found for the models predicting avoidance incongruence. Even after controlling for age, sex, pain intensity, pain interference, and pain duration, significant effects of time, between and within patient effects of psychological distress remained in the avoidance incongruence model.

### Exploratory Item Analyses

An exploratory regression analysis with a stepwise elimination strategy was conducted to determine which items of the incongruence questionnaire were associated with the best treatment outcomes in reducing psychological distress. Change scores for all INC-S scales, pain intensity, and pain interference were computed.

The first analysis including age, sex and pain duration as control variables was significant, *F*(3, 1896) = 8.12, *p* < 0.001. In a second analysis, psychological distress at pre-treatment, change in pain intensity and change in pain interference were added. The second analysis reached significance, *F*(6, 1893) = 505.43, *p* < 0.001 and the additional outcome variance explained 60.3%, *R*^2^ = 0.62, *F*_change_ (3, 1893) = 990.04, *p* < 0.001. This resulted in an adjusted *R*^2^ of 0.61. The third analysis included the change in the scale *Belief/Sense of Meaning* and was significant *F*(7, 1892) = 447.21, *p* < 0.001, the additional outcome variance explained 2.3%, *R*^2^ = 0.64, *F*_change_ (1, 1892) = 118.96, *p* < 0.001, and resulted in an adjusted *R*^2^ of 0.64. The fourth analysis included the change in the scale *Trust in Oneself* and was significant *F*(8, 1891) = 447.21, *p* < 0.001, the additional outcome variance explained 1.1%, *R*^2^ = 0.65, *F*_change_ (1, 1891) = 56.88, *p* < 0.001, and resulted in an adjusted *R*^2^ of 0.65. The final analysis shown in Table 8 included the change in the scale *Not Being Respected/Accepted* and was significant *F*(9, 1890) = 397.31, *p* < 0.001, the additional outcome variance explained 0.5%, *R*^2^ = 0.65, *F*_change_ (1, 1890) = 28.65, *p* < 0.001, and resulted in an adjusted *R*^2^ of 0.65. Multicollinearity was unproblematic in this model (Bowerman & O’Connell, 1990; Menard, 1995; Myers, 1990). Bonferroni correction was applied to correct for multiple comparison. Considering the 29 items in the explorative regression analysis, the adjusted *p* < 0.0017 marks statistical significance. Even after correcting for Bonferroni, the changes in the scales *Belief/Sense of Meaning, Trust in Oneself* and *Not Being Respected/Accepted* remained significant (*p* < 0.001).

**Table 8:** Exploratory regression analysis with stepwise elimination with mean psychological distress at post-treatment as outcome without Bonferroni correction

	*B*	*SE*	$\beta$	*t*
Constant	2.68	0.63		4.22***
Age	− 0.02	0.01	− 0.04	− 2.68**
Sex	0.03	0.24	< 0.01	0.11
Pain duration	0.02	0.01	0.03	1.96
Psychological distress HADS-D pre-treatment	0.77	0.01	0.76	55.36***
Change in pain intensity BPI	0.43	0.09	0.07	4.99***
Change in pain interference BPI	1.10	0.07	0.24	16.39***
Change in *Belief/Sense of Meaning*	0.78	0.09	0.12	8.54***
Change in *Trust in Oneself*	0.72	0.10	0.10	7.32***
Change in *Not Being Respected/Accepted*	0.50	0.09	0.08	5.35***

*B* unstandardized beta; *SE* standard error; $\beta$ standardized beta; *t t* value

*p < .05, **p < .01, ***p < .001

### Mediation Analyses

**Fig. 1:**
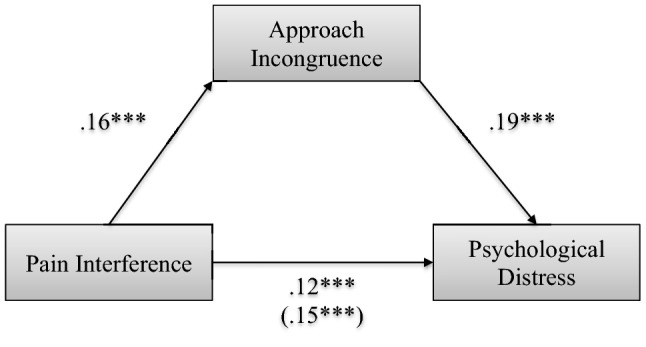
Mediation model of change in approach incongruence mediating the association between change in pain interference and change in psychological distress

Since both changes in pain interference and changes in approach and avoidance incongruence were associated with psychological distress at the end of interdisciplinary multimodal pain treatment, two separate mediation analyses for approach and avoidance incongruence were conducted to investigate the change of approach and/or avoidance incongruence across treatment as potential mediators of the effect of change in pain interference on change in psychological distress, respectively. Estimated change and intercept values of psychological distress, pain intensity, pain interference, approach, and avoidance incongruence were calculated. Age, sex, and pain duration were added as control covariates in the mediation analyses. Significant main effects (*p* < 0.001) of (1) change in pain interference on change in psychological distress and (2) change in pain interference on change in approach incongruence were found. The effect of pain interference change on change in psychological distress was significantly mediated by approach incongruence change (Fig. 1). The bootstrapped unstandardized indirect effect of approach incongruence change using 1000 bootstrapped samples was 0.03 (*p* < 0.001), and the 95% confidence interval ranged from 0.02 to 0.05. Change in avoidance incongruence did not significantly mediate the effect of change in pain interference on change in psychological distress. The results of the mediation analyses suggest that there is a mediation between the change in pain interference and change in psychological distress by the change in approach incongruence across treatment.

## Discussion

The aim of this study was to replicate the findings of Vincent et al. (2021) regarding the role of motivational incongruence in interdisciplinary multimodal pain treatment. Vincent et al. (2021) found significantly higher incongruence scores among patients with chronic pain than a healthy norm. Pain intensity, pain interference, psychological distress, and motive incongruence significantly reduced from intake to discharge. Furthermore, Vincent et al. (2021) found that only a reduction in approach incongruence was associated with reductions in psychological distress at discharge and vice versa. Moreover, the better satisfaction of approach motives seemed to mediate the effect of change in pain interference on change in psychological distress.

This study replicates the findings of Vincent et al. (2021) in that patients with chronic primary pain had significantly higher levels of incongruence in all sum scores (approach, avoidance, and total) as well as in most one-item comparisons with a healthy norm, with considerable variation in effect sizes (medium to high). In addition, significant reductions in pain intensity, pain interference, psychological distress, and motivational incongruence (approach, avoidance, and total) were found from pre- to post-treatment. Unlike Vincent et al. (2021), hierarchical linear models were used and revealed that reductions in both approach and avoidance incongruence were associated with the reduction of psychological distress, with significant within and between patient effects. Furthermore, psychological distress was associated with approach and avoidance incongruence post-treatment. Therefore, the HLM analyses did not suggest any causal direction of the association between motive incongruence and psychological distress. Exploratory regression analyses revealed that the better satisfaction of single motives like *Belief/Sense of Meaning, Trust in Oneself,* and the reduction of the feeling of *Not Being Respected/Accepted* might be the most promising targets for reducing psychological distress post-treatment. Like in Vincent et al. (2021), the better satisfaction of approach motives but not that of avoidance motives mediated the effect of change in pain interference on change in psychological distress.

Overall, patients with chronic primary pain reported higher incongruence scores in approach, avoidance, and total incongruence at intake compared to a healthy norm sample. This result supports the notion that insufficient motive satisfaction can be seen as a clinically relevant characteristic of patients with chronic primary pain to be considered in the assessment and treatment. As in the study of Vincent et al. (2021), exploratory single-item comparisons revealed that only the domains of incongruence regarding *Receiving Help*, *Altruism*, and *Hurting Others* were significantly higher in the norm sample. This difference may result from the patients currently receiving and accepting more help and support from clinical staff, their families, and their social environment. In contrast to Vincent et al. (2021), however, all single domains of motivational incongruence improved across treatment with small to medium effect sizes. Changes in the incongruence domains like *Education/Understanding*, A*ffiliation/Sociability, Appreciation/Approval, Separation/Being Alone*, or *Trust in Oneself* may be attributable to specific therapeutic interventions or to the inpatient treatment setting alone. Also, patients may learn during the treatment to better integrate pain into their lives, potentially impacting their sense of identity, their physical integrity, or their levels of goal achievement less, which in turn may be associated with less incongruence in domains such as *Status*, *Achievement/Performance*, *Belief/Sense of Meaning*, *Failure*, *Blame/Criticism*, and *Humiliation/Embarrassment* (Vervoort & Trost, 2017). In addition, better motive satisfaction and increased quality of life as well as well-being might relate to redefining and engaging in new and realistic goals in therapy (Esteve et al., 2018; Ramírez-Maestre et al., 2019).

The hierarchical linear models replicated the findings of Vincent et al. (2021) in that reductions in incongruence were associated with reductions in psychological distress at discharge. This finding supports the assumption of Consistency Theory that incongruence plays a crucial role in maintaining psychopathological symptoms and distress (Fries & Grawe, 2006). Other than Vincent et al. (2021), however, in the current study, not only the better satisfaction of approach motives but also that of avoidance motives was associated with reduced levels of psychological distress. Therefore, the increase of both desired experiences (indicating better need satisfaction) as well as the decrease in undesired experiences (assumedly resulting from fewer need violations) seem to be associated with less psychological distress, above and beyond improvements in pain-related factors. Significant within and between patient effects of incongruence on psychological distress suggest that both, the overall level of motive satisfaction regarding approach and avoidance goals and according changes over treatment may have substantial impacts on patients’ psychological distress. The finding that these effects remained when time effects and other pain-related factors were controlled for strengthens the notion that experiencing less psychological distress over treatment might be partly explained by experiencing less incongruence. However, the relationship between incongruence and psychological distress may be bidirectional in that reductions in psychological distress also entail reductions in incongruence. This would also be in line with previous findings (Brockmeyer et al., 2015; Kelly et al., 2015; Vincent et al., 2021), supporting the notion that psychological distress and mental disorders can be sources of incongruence themselves (Westermann et al., 2019).

Due to only approach motives significantly predicting reduced psychological distress post-treatment, Vincent et al. (2021) included only items of approach incongruence in their exploratory regression analysis. They found that increases in *Belief/Sense of Meaning, Self-Reward,* and *Control* were significantly associated with psychological distress. However, in this study, both approach and avoidance motives significantly predicted reduced psychological distress post-treatment and were included in the subsequent exploratory regression analyses. In these item-level analyses (each item in the short Incongruence Questionnaire INC-S, representing a scale in the larger INC), increases in *Belief/Sense of Meaning, Trust in Oneself,* and reductions regarding *Not Being Respected/Accepted* after treatment were associated with less psychological distress as treatment outcome, even after Bonferroni correction. Although these results are based on single items and should be interpreted with caution, these findings suggest which issues might require special attention in treating chronic primary pain, which should be studied in more detail in future research using the extended version of the INC questionnaire.

Given that both, changes in pain interference and changes in approach and avoidance incongruence were associated with psychological distress at the end of interdisciplinary multimodal pain treatment, two mediation models were tested. These two models tested the change of approach and avoidance incongruence across treatment as potential mediators of the effect of change in pain interference on change in psychological distress. The results showed that only changes in approach incongruence partially mediated the effect of change in pain interference on change in psychological distress, replicating the results of Vincent et al. (2021). Thus, having more gratifying experiences over treatment but not having less aversive experiences over therapy seem to predict less psychological distress post-treatment.

More generally, the findings of this study with patients with chronic primary pain in interdisciplinary multimodal pain treatment align with the assumptions of Consistency Theory (Grawe, 2004) in that patients` reduction in distress after treatment results from a better satisfaction of psychological needs via better motive satisfaction.

## Limitations and Future Research

Several limitations need to be addressed. Since this study only used the data of inpatients with chronic primary pain at one site who gave their consent to further use of their data, this study is not representative of all inpatients with chronic pain. Having only two measurement points at pre- and post-treatment, the current study design does not allow for any conclusions about the sustainability of effects or the direction of causality. Because interdisciplinary multimodal pain treatment includes different forms of therapy and is tailored individually for each patient, it is not possible to control for or manipulate all potential confounding variables. In addition, it is not possible to attribute changes post-treatment specifically to particular interventions in this setting, and some treatments may have had differential effects on the incongruence, psychological distress, and pain outcomes. To make predictive statements on the relationship between motivational incongruence, chronic pain, and the sustainability of the effects after treatment, future studies should have longitudinal designs. Furthermore, controlled and experimental longitudinal designs (e.g., cross-lagged analysis) are needed to draw conclusions on causality.

## Conclusion

Taken together, motivational incongruence seems to play a crucial role in the psychopathology and the treatment of chronic primary pain. Patients with chronic primary pain show significant differences in incongruence compared to healthy controls. Both psychological distress and motivational incongruence were reduced after interdisciplinary multimodal pain treatment. Furthermore, this replication study shows that both reductions in approach and avoidance incongruence are associated with reductions in psychological distress. As motivational incongruence can be reduced in inpatient psychotherapy (Berking et al., 2003), the findings of this study have potential consequences for treating chronic primary pain. Therefore, interdisciplinary multimodal pain treatment should focus on increasing desired experiences as expressions of need satisfaction and decreasing undesired experiences resulting from need violations. The results of the exploratory regression analysis indicate that especially interventions targeting the better satisfaction of motives like *Belief/Sense of Meaning, Trust in Oneself,* and the reduction of the feeling of *Not Being Respected/Accepted* may be the most promising targets for short-term interdisciplinary multimodal pain treatment. The present findings suggest that distress of patients with chronic primary pain may decrease over inpatient treatment due to functioning better in daily life, which may impact their trust in themselves, their feeling of being respected, and experiencing their lives as more meaningful again. In sum, reducing motivational incongruence among patients with chronic primary pain might help alleviate their psychological distress and improve their well-being despite their chronic pain.

## Data Availability

The data stems from inpatients with chronic primary pain who have received interdisciplinary multimodal pain treatment at the Inselspital Bern.
